# Achieving the vision of genomics to improve health for all requires a focus on diversity, equity and inclusion

**DOI:** 10.1007/s12687-025-00823-1

**Published:** 2025-07-21

**Authors:** Muin J. Khoury, Colleen M. McBride, Martina C. Cornel

**Affiliations:** 1 Department of Epidemiology, Rollins School of Public Health, Emory University, Atlanta, GA USA; 2 Department of Epidemiology, School of Public Health, University of Washington, Seattle, WA USA; 3 Department of Social Behavioral and Health Education Sciences, Emory University, Atlanta, GA USA; 4 Department of Human Genetics, Amsterdam University Medical Centre, Amsterdam, The Netherlands

In 2025, the United States is facing the possibility of substantial government budget cuts in biomedical research and public health (Wadman 2025; Rubin 2025, Ray 2025). Along with this is the termination of numerous established government programs and grants. Those being specifically scrutinized for cuts are research related to diversity, equity and inclusion (DEI) regardless of topic area (The White House 2025). DEI can have many meanings (Mallapaty 2025). Boundaries of what DEI research to improve human health would be allowed and what would not, are not well delineated, although several important programs have been cancelled. In a recent interview, the new NIH director Jay Bhattacharya distinguished between studies that look at the biological consequences of individual characteristics such as age, race and sex, which he said were important, and those involving political ideology which he said did not contribute to improving health (Kaiser 2025).

As the discussion around DEI continues to unfold, we are continuously reminded as scientists, physicians, and public health professionals that for genomic medicine to benefit all of humanity, “diversity”, “equity” and “inclusion” are core principles of and essential to research and practice.

The purpose of this editorial is to discuss DEI concepts in genomics research and practice and highlight selected examples of recommendations from the scientific community and global organizations on the importance of DEI in fulfilling the promise of health benefits of genomics in the 21st century. We end with a global call to action to help ensure that progress in genomics is not lost or reversed in the next decade.

We begin with the foundational concept of health equity, the notion that everyone has a fair and just opportunity to attain their highest level of health (Centers for Disease Control and Prevention 2024). In the context of genomics, we consider this to include fair and even access to genomic services such as testing and counseling, and unbiased implementation of genomic knowledge. (Jooma et al. 2019) To achieve health equity in genomics requires improved knowledge of the interaction among biological, social, and environmental factors in disease occurrence, and application of this knowledge (e.g. genetic testing) in health care services and disease prevention around the world.

Genomic research has made substantial contributions to our understanding of the genomic underpinnings of disease that in turn, have improved disease prevention, early detection, diagnostic accuracy, and therapeutic intervention. Over the last decade, efforts have accelerated to increase the inclusion of diverse populations to participate in genomics research (e.g. All of Us Research Program, Kozlowski et al. 2024). Diversity and inclusion efforts are essential to advance discovery and our ability to translate genomic discoveries to ensure that findings are generalizable to entire populations (Rebbeck et al. 2022). There are important reasons to include genetic diversity in research (Bentley et al. 2017) such as improved understanding of disease biology, more accurate matching of ancestrally diverse individuals with safe and effective prevention recommendations and treatments, and improved interpretation of genetic tests. A prominent recent example is the discovery of the *PCSK9* gene and its use in the treatment of familial hypercholesterolemia (FH) (Abifadel 2025). Genetic variants resulting in loss-of-function in *PCSK9* have been associated with reduced plasma levels of LDL-C and coronary heart disease. One of these variants is relatively common in the African-American population (approximately 2%) but relatively rare in populations of European ancestry (< 0.1%). The resulting discoveries paved the way for using PCSK9 inhibition to benefit all patients with FH regardless of their genetic ancestry (Rodriguez 2015).

Advances in genomics have led to an increasing number of evidence-based applications in clinical practice and disease prevention (commonly referred to as tier 1 applications, Khoury et al. 2022). Examples of tier 1 genomic applications in clinical practice today include newborn screening, as well as genetic testing for hereditary breast and ovarian cancer, Lynch syndrome, familial hypercholesterolemia (FH), hereditary hemochromatosis, and hypertrophic cardiomyopathy. For all these conditions, the overall implementation of current guidelines is suboptimal for the population as a whole, but most especially among women, people living in rural communities, racial and ethnic minority groups, people who are uninsured or underinsured, and those with lower education and income.

For these compelling reasons, we see numerous calls to action for a DEI agenda in genomic medicine (e.g. Popejoy 2016; Balogan and Olopade 2021; Editorial 2021, Rebbeck et al. 2022; Corpas et al. 2025) that will be derailed by DEI backlash against and deep cuts in any research that uses DEI nomenclature. In the decades after completion of the Human Genome Project (HGP), these calls focused on improving the rigor and applicability of basic science discoveries to have a tangible population health impact. In 2017, the *Journal of Community Genetics* published a special issue on inclusion of diverse populations in genomics research and health services: a scientific and health equity imperative (see editorial by Cornel and Bonham 2017) called for measurable milestones that require community engagement, to broaden sub-population inclusion, as well as active integration of genomics and health services across the globe. The collection highlighted the timeliness of addressing critical barriers as advances in sequencing technology and falling costs could make the integration of genomics a reality in the next decade.

Among several outstanding articles in this collection, Bentley et al. (2017) identified critical barriers to DEI in genomics research and health care. Important challenges to diversity include limited engagement by communities and research participants, preference of researchers to analyze data from well characterized and well-powered studies conducted in people of European ancestry, difficulties in conducting studies and obtaining funding due to relatively smaller sample sizes in subpopulations, limitations of genomic technologies in adequately capturing variation among diverse populations, analytic challenges in analyzing diversity, as well as the lagging diversity within the scientific community engaged in genomics research.

In 2022, the Centers for Disease Control and Prevention published its stakeholder-informed strategic planning effort in genomics and health equity emphasizing the need for leadership from public health organizations (Khoury et al. 2022) and the critical importance of access to genomics-informed interventions. Factors that influence access to genetic informed interventions are complex and will not be addressed by expanding availability. These services must also be acceptable to those who need them. The diverse contexts and preferences of rural communities, people with lower incomes, racial and ethnic groups will influence their acceptability. To date, uptake of available genetic services are lower among these groups, which can lead to lower rates of diagnosis, suboptimal care, and worse health outcomes. Khoury et al. (2022) suggested that health equity in genomics can be integrating into the essential public health functions of assessment, policy development, and assurance.

In 2022, the National Human Genome Research Institute (NHGRI) hosted a public workshop entitled *Future Directions of Genomics and Health Equity Workshop*, attended by over 300 individuals from various backgrounds. The workshop sought to leverage what has been learned in DEI that can inform direct action to advance the field. The recommendations from the workshop (Madden et al. 2024) were wide ranging and worthwhile repeating here:

1. Diversify the genomics workforce.
2. Address the lack of population diversity in genomics research and biobanks.
3. Assess how the lack of diversity in populations and communities comprising genomic research cohorts impacts health disparities.
4. Address outcomes important to all communities.
5. Address the inappropriate use of population descriptors in genomics research.
6. Increase the utilization of genomic markers rather than population descriptors in clinical algorithms.
7. Include contextual variables and diverse settings in genomics research.
8. Build partnerships with diverse communities to build trust, obtain feedback and conduct research in an equitable fashion.
9. Respect the views and autonomy of participants and communities.
10. Develop metrics of health equity and apply those metrics across genomics studies.

Interruptions in U.S. funding of genomics research has global consequences as well. In 2022, The World Health Organization’s Science Council issued its first report on “Accelerating Access to Genomics for Global Health” (WHO, 2022). The report made a strong case for less-resourced countries to gain access to such technologies. The report reminded us to prepare society for the complexities of this new field and ensure fair distribution among all countries. There were four broad recommendations that came out in the council’s report including:

1. Promotion of genomics through advocacy all member states of WHO especially low- and middle- income countries.
2. Provide expert council on best strategies for implementation of genomics methodologies at national and regional levels.
3. Strategic collaborations among entities engaged in genomics.
4. Attention to the ethical, legal, and social issues raised by genomics.

In 2024, the WHO genomics program was proposed, strategically designed to advocate for investments in the field, promote collaboration, overcome obstacles to implementation, and address inequitable access to genomic services (Ambrosino et al. 2024).

Taken together, the analyses and recommendations reviewed here represent a visionary way forward for achieving population health benefits of genomic discoveries. Today, we would like to reaffirm that for the ultimate success of genomic science to improving health for all, the *Journal of Community Genetics* and the *Public Health Genomics Journal* will continue to prioritize publications of DEI issues in genomic research and practice. The two journals will remain important platforms to discuss how to achieve the benefits of genomic knowledge for all. It is time to work together to clarify the fundamental concepts of DEI in genomics, medicine and public health. Revisiting the principles and actions that have been put forth by multiple organizations in the US and globally can help ensure that the decades-long investment in genomic research does not go to waste. We remain hopeful that we can fulfill the original aspirations of the Human Genome Project as we continue the long journey from gene discovery to improving health for all!
