# Correction: Achieving the vision of genomics to improve health for all requires a focus on diversity, equity and inclusion

**DOI:** 10.1007/s12687-025-00830-2

**Published:** 2025-09-04

**Authors:** Muin J. Khoury, Colleen M. McBride, Martina C. Cornel

**Affiliations:** 1 Department of Epidemiology, Rollins School of Public Health, Emory University, Atlanta, GA USA; 2 Department of Epidemiology, School of Public Health, University of Washington, Seattle, WA USA; 3 Department of Social Behavioral and Health Education Sciences, Emory University, Atlanta, GA USA; 4 Department of Human Genetics, Amsterdam University Medical Centre, Amsterdam, The Netherlands

**Correction: Journal of Community Genetics**

10.1007/s12687-025-00823-1

The article “Achieving the vision of genomics to improve health for all requires a focus on diversity, equity and inclusion”, written by Muin J. Khoury, Colleen M. McBride and Martina C. Cornel, was originally published under exclusive license to © The Author(s), under exclusive licence to Springer-Verlag GmbH Germany, part of Springer Nature 2025. As a result of the subsequent decision to publish the article under the open access model, the article’s copyright notice was changed on 13 August 2025 to © 2025 the Author(s). Published by Springer Nature in the Journal of Community Genetics and published by S. Karger AG in Public Health Genomics. This article is published under the Creative Commons CC-BY license and the article is now distributed under a Creative Commons Attribution 4.0 International License, which permits use, sharing, adaptation, distribution and reproduction in any medium or format, as long as you give appropriate credit to the original author(s) and the source, provide a link to the Creative Commons licence, and indicate if changes were made.

The images or other third party material in this article are included in the article’s Creative Commons licence, unless indicated otherwise in a credit line to the material. If material is not included in the article’s Creative Commons licence and your intended use is not permitted by statutory regulation or exceeds the permitted use, you will need to obtain permission directly from the copyright holder.

To view a copy of this licence, visit http://creativecommons.org/licenses/by/4.0.

Also, the second paragraph has been amended.

Furthermore, the joint publication note “This paper was jointly developed by Journal of Community Genetics and Public Health Genomics and jointly published by Springer Nature and S. Karger AG. The articles are identical except for minor stylistic and spelling differences in keeping with each journal’s style. Either citation can be used when citing this article.” has been added to the original article.

The original article was corrected.

